# Met/Val129 polymorphism of the full-length human prion protein dictates distinct pathways of amyloid formation

**DOI:** 10.1016/j.jbc.2022.102430

**Published:** 2022-08-28

**Authors:** Thomas Pauly, Najoua Bolakhrif, Jesko Kaiser, Luitgard Nagel-Steger, Lothar Gremer, Holger Gohlke, Dieter Willbold

**Affiliations:** 1 Institute of Biological Information Processing (IBI-7: Structural Biochemistry) and JuStruct: Jülich Center of Structural Biology, Forschungszentrum Jülich, Jülich, Germany; 2 Institut für Physikalische Biologie, Heinrich-Heine-Universität Düsseldorf, Düsseldorf, Germany; 3 Institute for Pharmaceutical and Medicinal Chemistry, Heinrich-Heine-Universität Düsseldorf, Düsseldorf, Germany; 4 Jülich Supercomputing Centre (JSC), Forschungszentrum Jülich GmbH, Jülich, Germany; 5 Institute of Bio- and Geosciences (IBG-4: Bioinformatics), Forschungszentrum Jülich GmbH, Jülich, Germany; 6 John von Neumann Institute for Computing (NIC), Forschungszentrum Jülich GmbH, Jülich, Germany

**Keywords:** aggregation, amyloid, analytical ultracentrifugation, creutzfeld–jakob disease, genetic polymorphism, prion, prion disease, CI, confidence interval, CNA, Constraint Network Analysis, MD, molecular dynamics, SASA, solvent-accessible surface area, SV, sedimentation velocity, ThT, thioflavin T

## Abstract

Methionine/valine polymorphism at position 129 of the human prion protein, huPrP, is tightly associated with the pathogenic phenotype, disease progress, and age of onset of neurodegenerative diseases such as Creutzfeldt–Jakob disease or Fatal Familial Insomnia. This raises the question of whether and how the amino acid type at position 129 influences the structural properties of huPrP, affecting its folding, stability, and amyloid formation behavior. Here, our detailed biophysical characterization of the 129M and 129V variants of recombinant full-length huPrP(23–230) by amyloid formation kinetics, CD spectroscopy, molecular dynamics simulations, and sedimentation velocity analysis reveals differences in their aggregation propensity and oligomer content, leading to deviating pathways for the conversion into amyloid at acidic pH. We determined that the 129M variant exhibits less secondary structure content before amyloid formation and higher resistance to thermal denaturation compared to the 129V variant, whereas the amyloid conformation of both variants shows similar thermal stability. Additionally, our molecular dynamics simulations and rigidity analyses at the atomistic level identify intramolecular interactions responsible for the enhanced monomer stability of the 129M variant, involving more frequent minimum distances between E196 and R156, forming a salt bridge. Removal of the N-terminal half of the 129M full-length variant diminishes its differences compared to the 129V full-length variant and highlights the relevance of the flexible N terminus in huPrP. Taken together, our findings provide insight into structural properties of huPrP and the effects of the amino acid identity at position 129 on amyloid formation behavior.

Transmissible spongiform encephalopathies are also known as prion diseases since they are based on the misfolding of the prion protein (1). The human prion protein (huPrP) is a membrane-bound glycoprotein, located mainly in nervous tissues, such as the brain and spinal cord. Two isoforms are known: the cellular form (huPrP^C^) is nonpathogenic, rich in α-helices (42%), and contains a single, small β-sheet (3%) (2). The other isoform, associated with the disease (huPrP^Sc^), contains 34% to 43% β-sheets and 20% to 30% α-helices (2, 3). Prions are defined as proteinaceous infectious particles (4), which in contrast to viruses lack any genetic information provided by nucleic acids. The structural conversion from huPrP^C^ into huPrP^Sc^ and aggregation into toxic oligomers and fibrils is governed by autocatalytic processes (5). Recently published high resolution structures of PrP amyloid fibrils by cryo-EM revealed a typical β-sheet structure (6, 7). Several neurodegenerative diseases such as Creutzfeldt–Jakob disease, Fatal Familiar Insomnia, Gerstmann-Sträussler-Scheinker syndrome, and Kuru are human prion diseases and associated with these amyloid structures.

The well-known methionine/valine polymorphism at position 129 appears in the cellular conformation at the beginning of the first β-strand (Fig. 1*A*). About 51% of the human population are heterozygous at position 129, 12% have a genotype of valine/valine, and the remaining 37% a genotype of methionine/methionine (8). This methionine/valine polymorphism is associated with the age of onset, the disease progress, and which pathogenic phenotype is developed in patients (9, 10, 11, 12). The polymorphism at position 129 raises the question of how it determines the pathogenic roles, especially with respect to the aggregation behavior during the conversion of huPrP^C^ into huPrP^Sc^. In several studies, properties of the amyloid fibril structure (13) and unfolded state (14, 15) of different prion protein variants had been investigated, revealing not only different amyloid fibril morphologies (16) within these variants and other mutations (17) but also further requirements for fibril formation such as a disulfide bond (15). This work presents a detailed biophysical characterization of full-length huPrP(23–230) for both variants and a comparison with a shorter construct, lacking the unstructured N-terminal region, huPrP(121–230) for the 129M variant. Understanding the impact of a single amino acid residue exchange on the propensity of huPrP to convert into the pathogenic amyloid structure *in vitro* will help to gain insights into the pathomechanisms of prion diseases.

**Figure 1:**
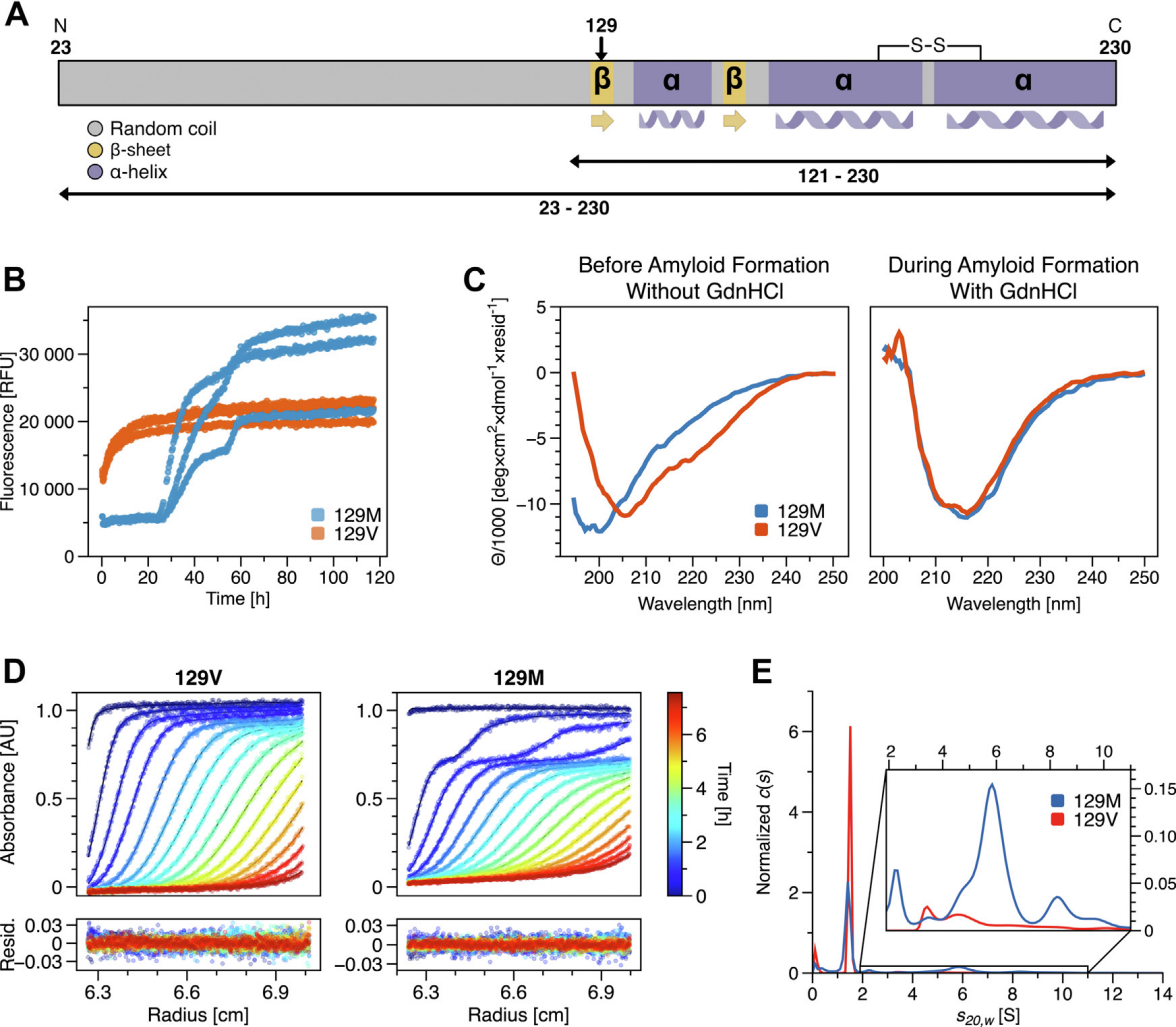
**Aggregation behavior of full-length huPrP (23–230).***A*, scheme of full-length huPrP(23–230) secondary structure elements. huPrP(23–230) comprises the unstructured N-terminal half and structured C-terminal domain, containing a disulfide bond between C179 and C214; huPrP(121–230) lacks the unstructured N-terminal region. *B*, amyloid formation kinetics are monitored by ThT fluorescence for triplicates of the 129M variant (*blue*) and 129V variant (*red*). 15 μM huPrP(23–230) was incubated with 0.5 M GdnHCl for destabilization and initialization of amyloid formation. *C*, secondary structure as represented in CD spectra of 10 μM huPrP(23–230) without (left) and with 0.5 M GdnHCl (right) after 5 h incubation for both variants. The incubation conditions are identical to amyloid formation kinetics. *D*, SV analysis of 7.5 μM of both variants without GdnHCl. Raw data from SV analysis with fitted Lamm equation solutions from *c*(*s*) model are color coded for the duration of sedimentation. *E*, the result of data fitting is an *s*-value distribution. SV; sedimentation velocity; ThT, thioflavin T.

Since the recombinantly produced full-length huPrP(23–230) does not aggregate spontaneously at physiological pH within manageable time ranges, we established an *in vitro* conversion system at acidic pH with additional destabilization by guanidine hydrochloride (GdnHCl). Amyloid formation kinetics monitored by the fluorescence dye thioflavin T (ThT) was used to examine aggregation pathways. CD spectroscopy was used to determine the secondary structure content before and during the amyloid formation process as well as the thermal stability before and after amyloid formation. Additionally, molecular dynamics (MD) simulations and rigidity analyses were performed to investigate the stability of both variants in detail. Sedimentation velocity (SV) analysis was performed to investigate the monomeric and oligomeric states before amyloid formation.

## Results

Differences in amyloid formation kinetics of both variants monitored by ThT fluorescence are presented in Fig. 1*B*. At pH 2.0, we used 0.5 M GdnHCl for an additional weak destabilization of 15 μM huPrP(23–230) to accelerate conversion into an amyloid structure (different conditions for huPrP and GdnHCl concentrations were tested, see Figs. S2–S4). Strikingly, both variants behave distinctly in the ThT kinetic assay. However, both variants have in common that neither amyloid formation nor an initial fluorescence plateau occurred in the controls without GdnHCl (Fig. S2), indicating no conversion of huPrP(23–230) without destabilization within the observation time. The 129M variant shows a more complex amyloid formation behavior, resembling kinetics with multiple phases. The kinetics to reach the final plateau are retarded compared to the 129V variant and passing through two interim plateaus. The 129M variant shows clear initial ThT fluorescence identical to the triplicates. The first steep increase starts reproducibly at about 25 h. Differences are observed in the duration of the single growth phases, their number and heights of interim and final plateaus. The time point of the second increase is approximately 55 h. Overall, the reproducibility within the triplicates is low. In contrast, the 129V variant shows an immediate steep ThT fluorescence increase without any lag phase. The amyloid formation of the 129V variant exhibits a single phase aggregation behavior. The reproducibility among the triplicates is high. Nevertheless, despite the obvious differences, both variants result in rather similar ThT fluorescence after 120 h. Measuring the concentration of the soluble fraction after 120 h revealed 72.5% and 84.1% of aggregated protein for the 129M and 129V variants, respectively (Fig. S5).

CD spectra were measured to determine the secondary structure content of huPrP (Fig. 1*C*). Here, 10 μM huPrP(23–230) without GdnHCl before amyloid formation at pH 2 was analyzed for both variants (Fig. 1*C*, left). Since CD is a bulk method, the signal represents the weighted average in secondary structure content for the sample. Oligomeric species with different monomer conformations might contribute to the measured spectra. For both variants, we observed CD spectra with a shape resembling a mixture of mainly α-helical and random coil structure. The position of the first minimum differs; it is at about 200 nm for the 129M variant and about 207 nm for the 129V variant. Additionally, a more positive signal between about 207 and 240 nm is observed for the 129M variant, suggesting a somewhat higher content of random coil structure. The secondary structure content was also investigated at time points during the amyloid formation process in the presence of 0.5 M GdnHCl (Fig. 1*C*, right, Fig. S7). Five hours after the addition of GdnHCl, a horizontal shift of the minimum from 200 nm to approximately 218 nm is observed for both variants, indicating an increase in β-strand conformation. This shift barely changes for longer incubation time but a drop of the peak signal at 218 nm was observed together with a loss of the accompanying UV absorbance signal (Fig. S7), so that one can assume a loss of soluble protein due to aggregation (18). These results support the conversion into amyloid for both variants. Although the amyloid formation behavior of the 129M variant seems retarded and follows a more complex and time-consuming mechanism, the difference in amyloid formation behavior between both variants is not reflected in the secondary structure of converted forms.

SV analysis was performed to characterize the hydrodynamic properties of both variants at pH 2 and examine the molecular assemblies present before amyloid formation (Fig. 1, *D* and *E*). These conditions can be referred to as starting conditions before GdnHCl is added for destabilization and initialization of amyloid formation. Fig. 1*D* shows raw data as sedimentation profiles with fitted Lamm equation solutions. The resulting distribution of standardized sedimentation coefficients (*s*_*20,w*_) presents differences in the degree of oligomerization between both variants (Fig. 1*E*). Molar mass is calculated based on the globally determined frictional ratio (*f/f*_0_), which is proportional to the hydrodynamic radius. At pH 2, *f/f*_0_ was about 2.2 for both variants, indicating an elongated shape. The 129M variant shows a faster sedimenting oligomer boundary, representing 31.6% of the total signal and corresponding to *s*-values between 2 and 15 S (potentially dimers to 15-mers). The remaining 68.3% of the sample can be assigned to the monomer state at 1.41 S. In contrast, most of the 129V variant is monomeric (93.1%) at 1.49 S and only 6.8% are oligomers between ∼2 and 15 S. A closer examination of the oligomer distribution yields significant differences for the smallest detectable species. A distinct peak can be assigned at 2.3 S for the 129M variant with a molar mass corresponding to a dimer. The smallest oligomer of the 129V variant can be assigned to a peak at 3.5 S with a molar mass appropriate for a trimer. The peak at 3.5 S can also be found in the distribution of the 129M variant. The 129M variant is more susceptible to oligomerization than the 129V variant and forms dimers at pH 2.0 without GdnHCl. The addition of GdnHCl had no impact on the monomer sedimentation coefficient corrected for increased density and viscosity of both variants. This indicates a similar shape of the monomer both in the presence and absence of GdnHCl, and thus, only minor changes in the fold induced by GdnHCl facilitate amyloid formation. A similar effect was published before with urea as a denaturant (18).

**Figure 2:**
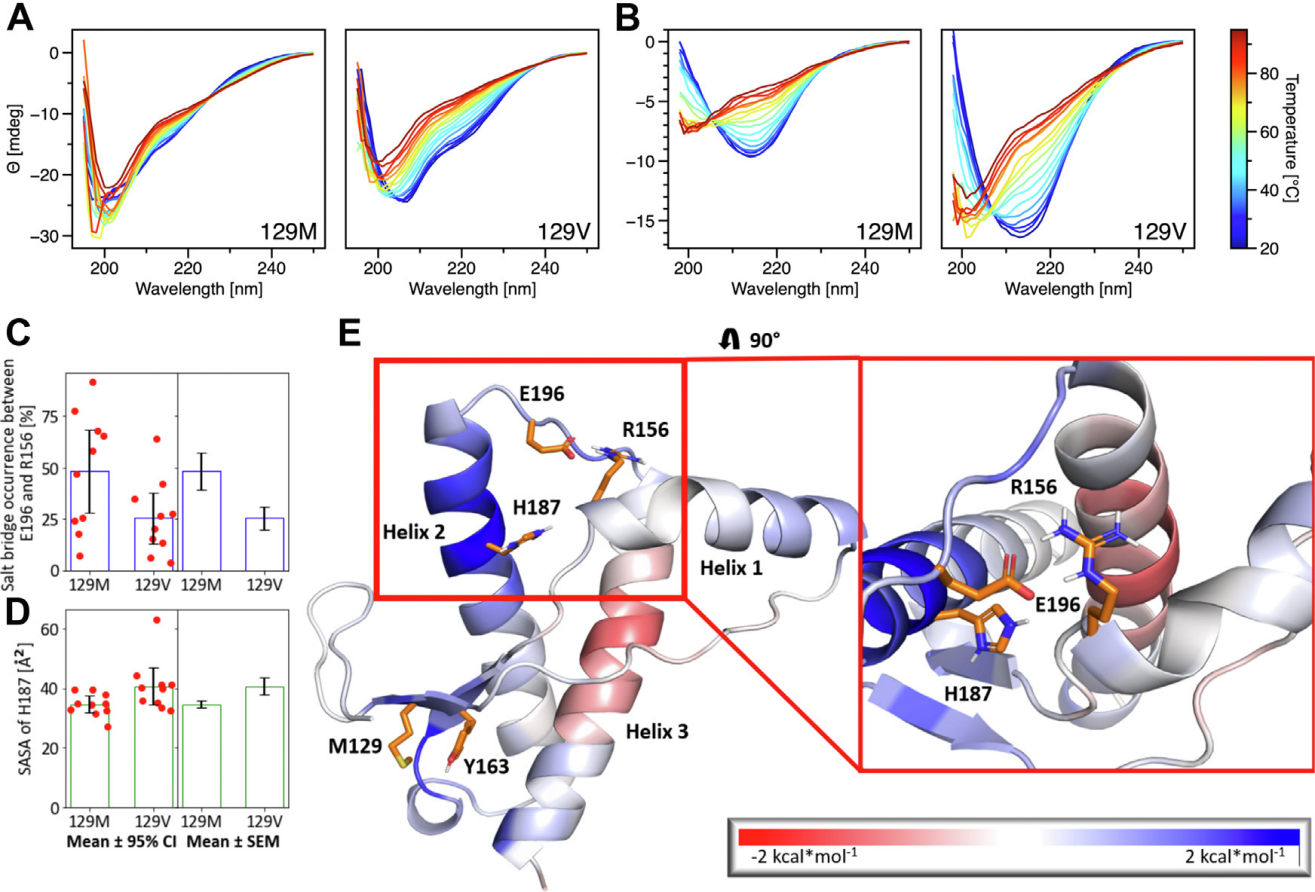
**Stability of huPrP.***A*, thermal denaturation over a temperature range from 20 °C to 95 °C before amyloid formation at pH 2 and (*B*) after amyloid formation at the end of kinetic experiments (after 120 h) of both variants of full-length huPrP(23–230). CD spectra of 10 μM of the 129M (*A*, left) and 129V (*A*, right) variant. CD spectra of end products from amyloid formation kinetics of 20 μM of the 129M (*B*, left) and 129V (*B*, right) variant. *C*, occurrence of salt bridge formation between E196 and R156 and (*D*) SASA values of H187 during MD simulations for both polymorphs. In (*C*) and (*D*), the mean ± 95% CI and all data points (left), and the mean ± SEM (right) are shown; the two mean values differ significantly, respectively (see text). *E*, differences of the residue-wise structural stability as determined from *rc*_*ij,neighbor*_ maps from CNA mapped onto the nonamyloid conformation of huPrP(118–224). *Blue* indicates that the respective area is more stable in the 129M variant. CI, confidence interval; MD, molecular dynamics; SASA, solvent-accessible surface area.

To probe differences in thermal stability, CD spectra of 10 μM huPrP(23–230) for each variant were recorded over a temperature range from 20 °C to 95 °C (Fig. 2*A*). The same stability test was performed for the amyloid conformation obtained after 120 h incubation (Fig. 2*B*). Samples with amyloid conformation were taken from kinetic experiments. Before amyloid formation, the 129M variant shows at 20 °C a spectrum that indicates higher random coil content than the 129V variant, and the spectrum only weakly changes with increasing temperature (Fig. 2*A*, left). The CD spectrum before amyloid formation of the 129V variant at 20 °C exhibits more initial secondary structure and changes clearly during temperature increase (Fig. 2*A*, right). For both variants, a rise of the CD signal at about 210 nm is observed during temperature increase, indicating an increase in random coil structure (19). The CD spectra at 20 °C of the amyloid conformation show a high content of β-sheet structure, which is similar for both variants (Fig. 2*B*). The amyloid structure of both variants exhibits clearly a structural change upon temperature increase. The final spectrum at 95 °C resembles the secondary structure content of samples before amyloid formation, indicating reversibility of the amyloid structure for both variants at pH 2. Note that the signal of the amyloid conformation is higher for the 129V variant, which hints at a larger amount of amyloid structures at the end of kinetic experiments, in agreement with the concentration left in the soluble fraction (Fig. S5). The lower impact of thermal denaturation on the 129M variant before amyloid formation compared to the 129V variant may explain the retarded conversion into amyloid structure observed in kinetic experiments. Despite the differences between both variants in amyloid formation kinetics, the final products present similar structures and thermal stabilities.

To investigate underlying reasons at the atomistic level for the different thermostability of the variants before amyloid formation, we performed 10 replicas of 1 μs long all-atom MD simulations for the protein fold at pH 2 of PrP(118–224) of both variants. Subsequently, we performed rigidity analyses using Constraint Network Analysis (CNA) (20) to compute the chemical potential energy averaged over the conformational ensemble, which correlates with the thermostability of proteins (20, 21). Accordingly, the folded domain of the 129M variant is more stable (*E*_CNA_ = –1003.3 kcal/mol) than the 129V variant (*E*_CNA_ = −966.1 kcal/mol). To elucidate the molecular interactions that lead to this enhanced stability, we computed the differences in the neighbor stability maps (*rc*_*ij,neighbor*_) generated by CNA (20). *rc*_*ij,neighbor*_ indicates if a rigid contact between two residues is weaker or stronger. A contact is considered to be rigid when both residues belong to the same rigid cluster along the constraint dilution trajectory (20, 22) and therefore indicates the structural stability of the involved residue pair. M129 stabilizes the region located close to the substitution site. The longer side chain of methionine allows more apolar interactions with neighboring amino acids. Furthermore, methionine can form interactions with the aromatic side chain of Y163 (Fig. 2*E*), further stabilizing the 129M variant (23). M129 also has an allosteric stabilizing impact reaching over 20 Å to the C-terminal part of helix2 (Fig. 2*E*). This region, especially H187, has been described to play a role in stabilizing the protein (24, 25). Because of a p*K*_a_ value of about 5, H187 is protonated at acidic but not at physiological pH (26). The protonated form of H187 can disrupt the salt bridge between R156 and E196, which acts as an anchor between helix1 and helix2/helix3, by interacting with E196 (24). Furthermore, the solvent-accessible surface area (SASA) of H187 has been reported to increase in partially unfolded structures because H187 initially forms a hydrophobic core located between helix2 and helix3 with the residues P158, F198, and M206; the protonation of H187 decreases the hydrophobicity and potentially leads to a destabilization of this hydrophobic core (25). Thus, we analyzed the mean values of the percentage of salt bridge formation between E196 and R156 and the SASA of H187 during our MD simulations. The results show a higher percentage of salt bridge formation for the 129M polymorph (48.2 ± 9.0%, 95% confidence interval (CI): 27.9% - 68.5%) in comparison to the 129V polymorph (26.0 ± 5.5%, 95% CI: 13.5% - 38.5%). The mean values for both polymorphs differ significantly (*p* = 0.02; unpaired *t* test) (Fig. 2*C*). Furthermore, the SASA of H187 is significantly decreased in the 129M polymorph (34.6 ± 1.2 Å^2^, 95% CI: 31.8 Å^2^ – 37.3 Å^2^) compared to the 129V polymorph (40.7 ± 2.8 Å^2^, 95% CI 34.4 Å^2^ – 47.0 Å^2^) (*p* = 0.03; unpaired *t* test) (Fig. 2*D*), confirming the increased structural stability in this region for huPrP (118–224) (Fig. 2*E*). The lower stability of the investigated 129V variant might favor amyloid formation by lowering energetic barriers considering the single amino acid exchange at position 129 (27).

**Figure 3:**
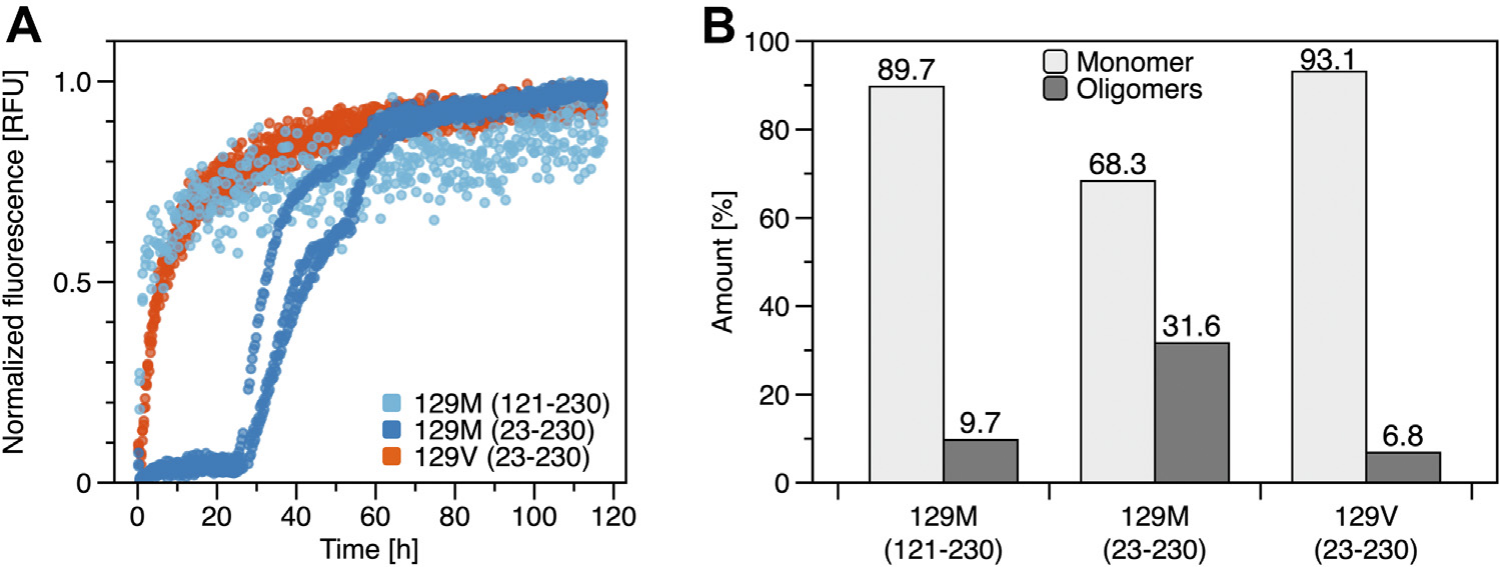
**Relevance of the unstructured N-terminal region.***A*, amyloid formation kinetics monitored by ThT fluorescence of the 129M variant huPrP(121–230) lacking the unstructured N-terminal region (*light blue*). Kinetics for full-length huPrP(23–230) 129M variant (*dark blue*) and 129V variant (*red*) from Fig. 1*B* is shown again for comparison. Kinetic data are normalized to 1 for the highest signal. 15 μM huPrP was incubated with 0.5 M GdnHCl for destabilization and initialization of amyloid formation. *B*, amount of monomer and oligomers of 129M and 129V variants huPrP(23–230) and the 129M variant huPrP(121–230). The amount is the result of integration of *c*(*s*) distributions from SV analysis of 7.5 μM huPrP without GdnHCl at pH 2 (Fig. S6). ThT, thioflavin T.

Studies investigating the polymorphism at position 129 generally used a shorter construct of huPrP, lacking the unstructured N-terminal domain (28, 29). To test the effect of the unstructured N-terminal domain on aggregation kinetics and oligomer formation, we compared a shorter construct of the 129M variant without the N-terminal half, huPrP(121–230), to our full-length proteins (Fig. 3). The lack of the unstructured N-terminal domain of the 129M variant significantly alters the amyloid formation kinetics (Fig. 3*A*). The shorter construct of the 129M variant does not present multiphasic, complex kinetics but shows an immediate onset of amyloid formation as already observed for the full-length 129V variant. SV experiments report a lower degree of oligomerization and a higher monomer content for huPrP(121–230) of the 129M variant compared to full-length huPrP(23–230) (Figs. 3*B* and S6). The monomer and oligomer content of the shorter 129M variant is similar to the full-length 129V variant, which agrees with the similarities found in amyloid formation kinetics. We conclude that the presence of the unstructured N-terminal region affects the structural properties of the protein, leading to significant changes in oligomerization and hence in amyloid formation kinetics.

## Discussion

In summary, our study revealed clear differences in the pathway of amyloid formation at pH 2 of full-length huPrP(23–230) associated with the single Met/Val amino acid exchange at position 129, which provide insights into possible mechanisms underlying prion diseases. Additionally, we observed a significant impact of the unstructured, 98 amino acid long N-terminal region on the aggregation propensity of the 129M variant, superimposing the direct effect of the polymorphism at position 129. The deviating stability of both variants of huPrP(118–224) observed during MD simulations suggests a different unfolding behavior based on this polymorphism. We observed an initial ThT fluorescence plateau in kinetic measurements upon the addition of GdnHCl for the 129M variant. The early ThT signal supports an immediate formation of oligomers with partial binding sites for ThT, which was already reported for the 129M variant (30). After the initial formation of aggregates, no further decrease of the soluble fraction was observed. As a consequence, the increase in ThT fluorescence leading to interim and final plateaus observed in the multiphasic kinetics of the 129M variant originates from structural rearrangements of already existing larger oligomers. A stronger tendency for oligomerization of the 129M polymorphism was also observed in SV experiments without GdnHCl. The capability of larger oligomers to assemble into amyloid fibrils was previously shown for ovine PrP (31). We conclude that the amyloid formation pathway for the 129M variant is different to the 129V variant and characterized by the presence of oligomeric intermediates undergoing further structural conversion. The increased population of non-native states but not yet fully amyloid structure for the 129M variant might explain differences in pathology associated with this polymorphism. Interestingly, the existence of a detectable amount of dimers was already reported to interfere with the conversion of huPrP^C^ into huPrP^Sc^ (32). Observed *s*-values appropriate for dimers in SV experiments were exclusively found for the 129M variant, providing a further explanation for higher resistance to amyloid formation and more complex kinetics.

The differences in structure and global stability of huPrP^C^ as a consequence of the polymorphism at position 129 were already investigated in NMR studies (28). It was reported that the polymorphism at position 129 in a shorter construct excluding the unstructured N-terminal domain, huPrP(90–230), neither impacts on structure nor stability. It should be noted that the polymorphism was investigated at pH 5.5. At this pH, H187 is not completely in a protonated state. It was further reported that the polymorphism might affect amyloid formation kinetics or the formation of intermediates on the pathway of conversion of huPrP^C^ into huPrP^Sc^. The overall structure of the C-terminal globular domain of huPrP(125–228) is not affected by constructs of different lengths but a transient contact between the flexible, unstructured N-terminal region and the globular C-terminal domain was observed in NMR studies (33). The length of the unstructured, N-terminal region affects helix2(187–193) and helix3(219–226) in the globular domain. Hence, the usage of full-length huPrP(23–230) leads to a more complete picture of the underlying structural rearrangements by including the complete, flexible N terminus.

This study aimed to investigate the differences between the two variants with regard to their aggregation propensity and possible amyloid formation pathways. We concluded an oligomer-mediated pathway for the 129M variant but could only observe such behavior for the full-length variant. The described data result as a consequence of both described effects, the exchange of a single amino acid as well as the presence of the N-terminal half, however, the effect of the single amino acid exchange seems to be superimposed by the depletion of the 98 amino acid long N-terminal half. Our results emphasize the relevance of the considered full-length huPrP(23–230) construct to perceive different aggregation propensities.

## Experimental procedures

All experiments were performed at pH 2 in 10 mM aqueous HCl.

### Expression and purification of human prion protein

**SI Figure 1:**
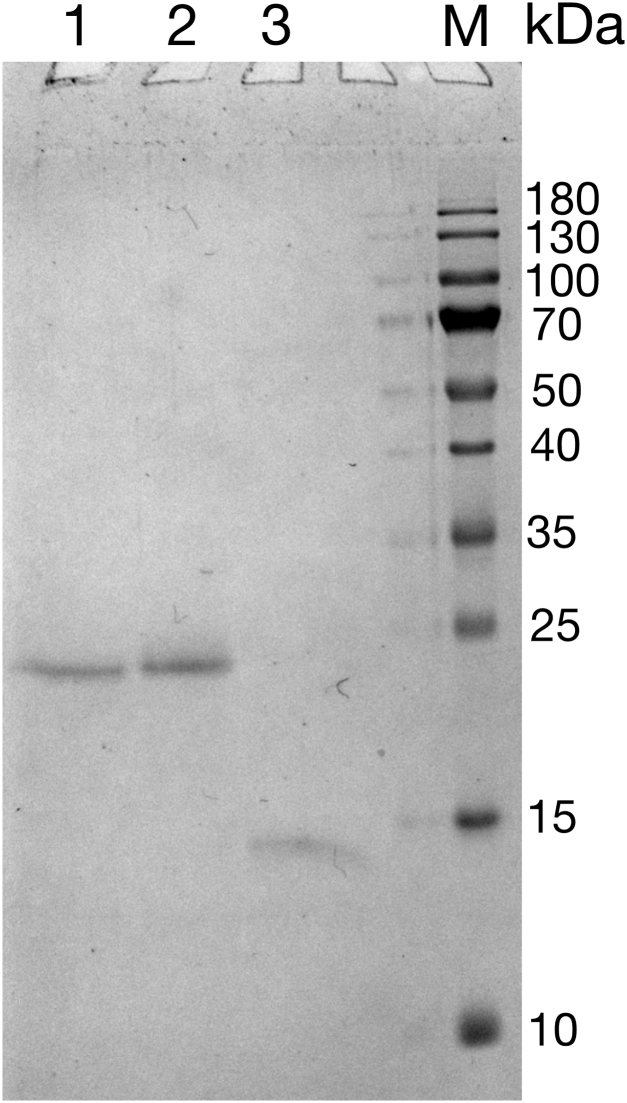


**SI Figure 2:**
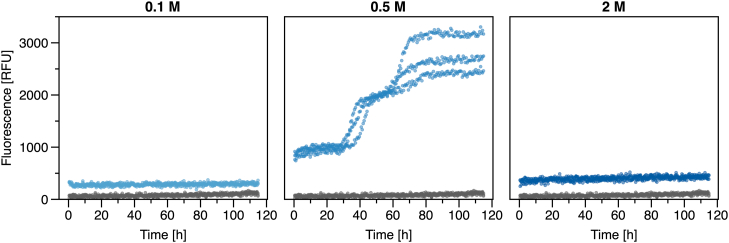


**SI Figure 3:**
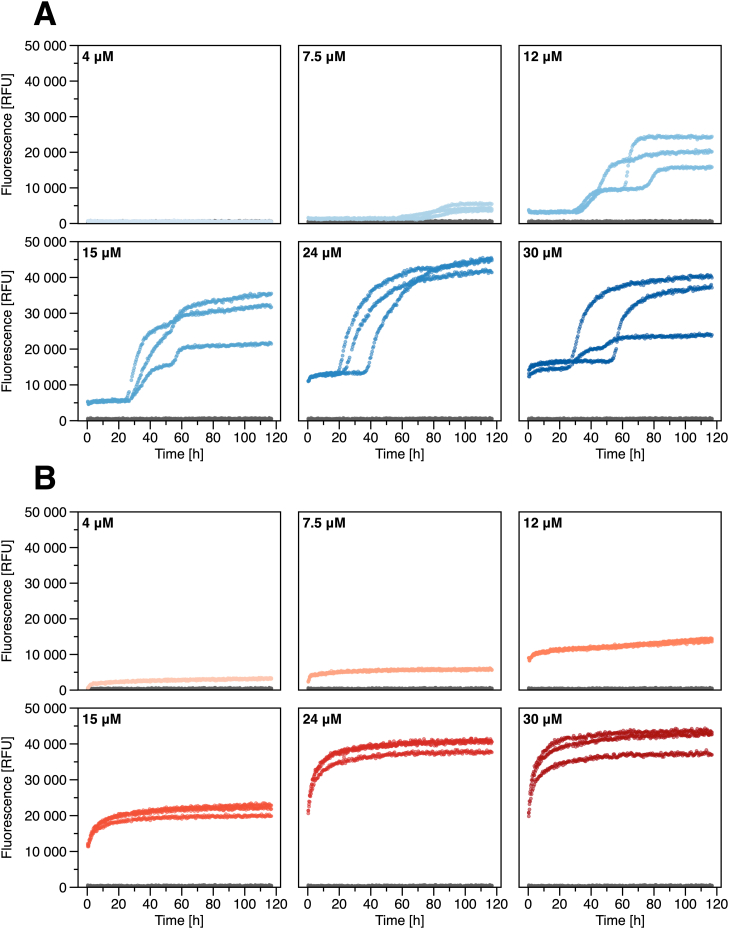


**SI Figure 4:**
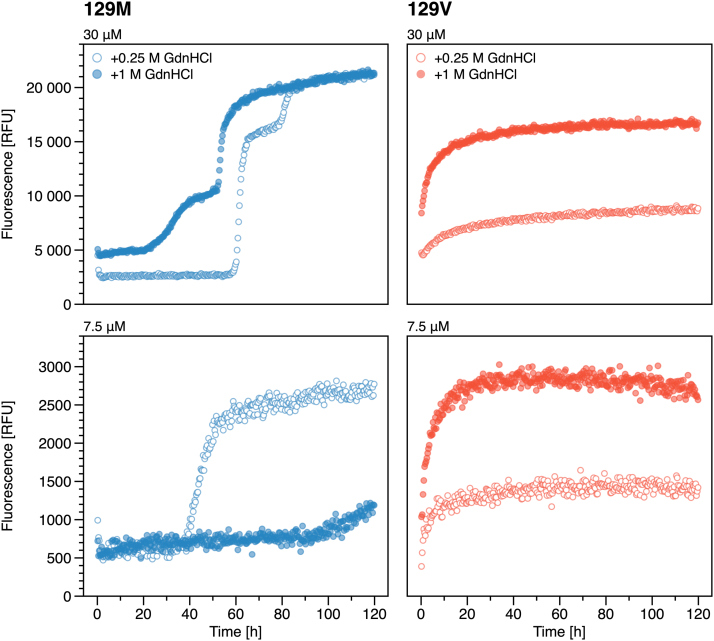


**SI Figure 5:**
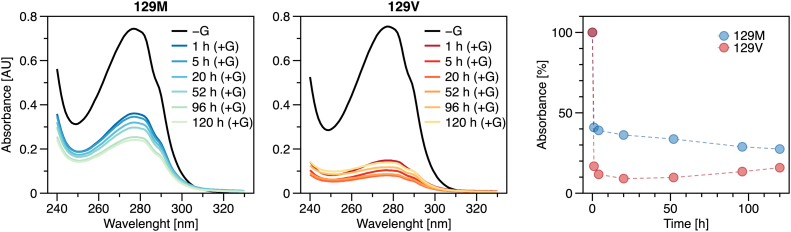


**SI Figure 6:**
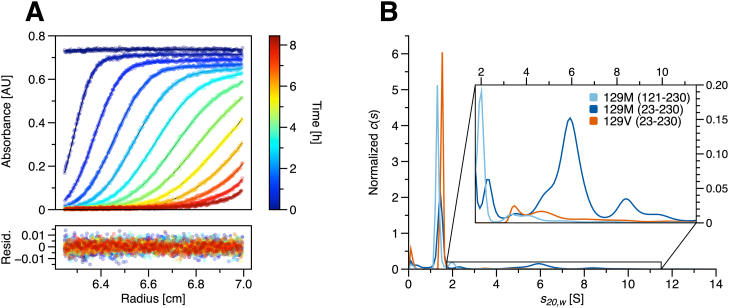


**SI Figure 7:**
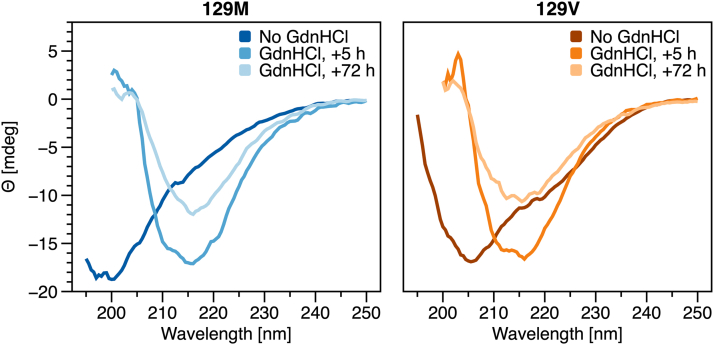


Both recombinant huPrP(23–230) variants 129M and 129V as well as the huPrP(121–230) 129M variant were expressed and purified as previously described (34). The protein folding following this purification protocol and acidic pH was previously confirmed by NMR (34). Final sample purity was confirmed by SDS-PAGE (Fig. S1).

### Amyloid formation kinetics

Amyloid formation kinetics of both huPrP(23–230) variants and huPrP(121–230) 129M variant were monitored by ThT fluorescence using a plate reader (BMG). ThT fluorescence was measured by excitation at 445 nm and detection at 485 nm. About 15 and 20 μM huPrP with 0.5 M GdnHCl and 30 μM ThT were measured. The measurements were performed in triplicates in a 96-well plate (No. 3881, Corning) sealed with a plastic film at 37 °C and continuous shaking at 300 rpm.

### CD spectroscopy

CD spectra of 10 μM huPrP(23–230) for both variants were recorded in a Jasco J-815 (Jasco) spectropolarimeter at 20 °C without GdnHCl and after 5 h in the presence of 0.5 M GdnHCl. Thermal stability was investigated over a temperature range from 20 °C to 95 °C. The thermal stability was investigated before amyloid formation of 10 μM huPrP(23–230). To investigate the thermal stability of the amyloid conformation, 100 μl were taken from amyloid formation kinetics of 20 μM huPrP(23–230) after 120 h and centrifuged at 15,000×*g* for 25 min and 20 °C. The pelleted amyloid structures were dissolved in 100 μl 10 mM HCl (pH 2). A quartz glass cuvette with 1 mm path length was used. Spectra were measured at 50 nm/min with 2 nm bandwidth and 4 s digital integration time. Spectral resolution was 1 nm and temperature resolution was 5 °C. The temperature was increased with 2 °C/min and 30 s waiting time before measurement. Ten accumulations were measured for each spectrum.

### Analytical ultracentrifugation

SV experiments were performed in an analytical ultracentrifuge Proteome Lab XL-A (Beckman-Coulter). Experiments included 7.5 μM huPrP. Samples were measured in standard double sector cells (Titanium) with an optical path length of 12 mm using an An-60Ti rotor. Temperature was set to 20 °C. The speed was 60,000 rpm corresponding to 262,000×*g*. Data analysis was performed using a continuous distribution Lamm equation model, *c*(*s*), implemented in the software Sedfit (version 16p35; https://sedfitsedphat.github.io) (35).

### MD simulations

**SI Figure 8:**
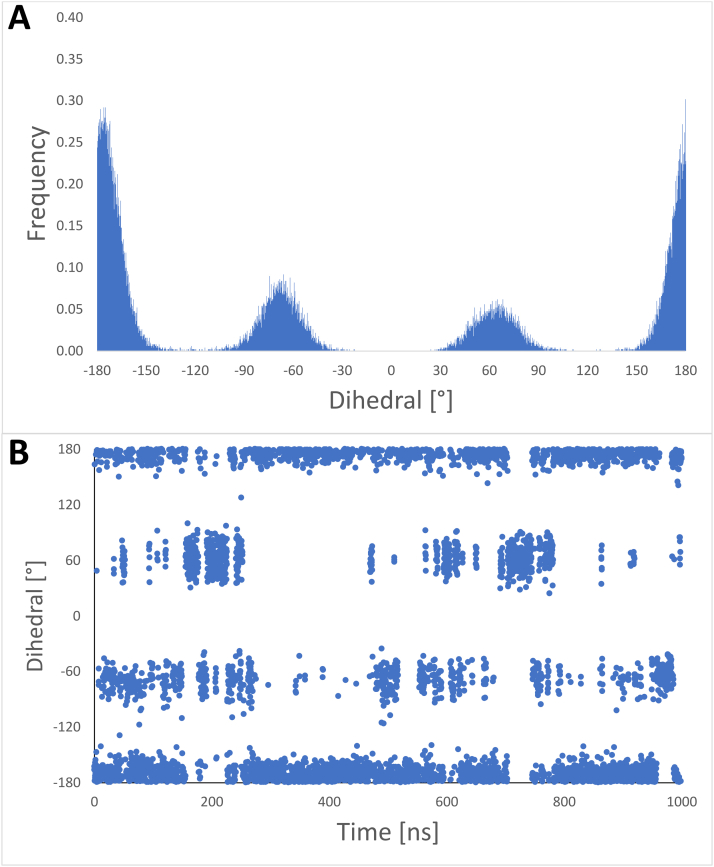


Structural information for PrP(118–224) 129M was taken from Protein Data Bank entry 4N9O (36). For the 129V polymorph, the amino acid at position 129 was mutated to valine using MOE, version 2019.01 (37). We selected the energetically most favored rotamer and subsequently minimized the side chain; both polymorphs were protonated according to pH 2, and the N and C termini were capped with NME and ACE, respectively, using MOE, version 2019.01 (37). With a predicted p*K*a value of 2.09, E196 can be protonated or deprotonated at pH 2 (38). As E196 forms a salt bridge with R156 in our starting structure, which stabilizes a negative charge at E196, it is likely that E196 is deprotonated in the starting structure. The polymorphs were then neutralized using Cl^-^ as counter ions and solvated in an octahedral box of OPC water (39) with a minimal water shell of 12 Å around the protein. The Amber package of MD simulation software (40) and the ff19SB force field (41) were used to perform MD simulations. For further details of our simulation methods, see supporting information and Fig. S8.

### Structural analyses

To ensure that the starting structures of both polymorphs do not influence the results, we removed the first 500 ns of MD simulations prior to the analyses. Every 5 ns, a frame was extracted from the trajectories using CPPTRAJ (42), and counter ions and water molecules were stripped. From the protein conformations, neighbor stability maps *rc*_*ij,neighbor*_ were calculated using CNA (20). CNA is a software package that functions as frontend and backend for the FIRST software (https://kuhnlab.natsci.msu.edu/software/proflex/) and helps to analyze structural features critical for protein stability; neighbor stability maps are derived from all extracted trajectories along the simulation and contain information about the persistence of rigid contacts between pairs of residues (20, 43). In order to exclude pairs of structurally non-neighboring residues, only interactions were considered in *rc*_*ij,neighbor*_, where at least one of the pairs of heavy atoms of the residue pair *R*_(*i*,*j*)_ is separated by less than 5 Å. The chemical potential energy *E*_*CNA*_, a measure for thermostability, was calculated according to Equation 1 as done previously (44).

(1) $$E_{CNA}=\sum_{i}^{n}\sum_{j>i}^{n}rc_{ij,neighbor}$$

The distance between the carboxy oxygens of E196 and the side chain nitrogen atoms of R156 was calculated using the *nativecontacts mindist* command as implemented in CPPTRAJ (42). We consider a salt bridge formed if the distance between respective charged heavy atoms is < 4 Å (45). The SASA of H187 was analyzed using the *surf* command as implemented in CPPTRAJ (42). We calculated the one-sided *t* test for both measurements with the null hypothesis that the values of the two stability-indicating measurements are in favor of the more stable 129M polymorph.

## Data availability

All described data are included in this article.

## Supporting information

This article contains supporting information (42, 46, 47).

## Conflict of interest

The authors declare that they have no conflicts of interest with the contents of this article.

## References

[1] Prusiner S.B., McKinley M.P., Groth D.F., Bowman K.A., Mock N.I., Cochran S.P. (1981). Scrapie agent contains a hydrophobic protein. Proc. Natl. Acad. Sci..

[2] Pan K.-M., Baldwin M., Nguyen J., Gasset M., Serban A., Groth D. (1993). Conversion of alpha-helices into beta-sheets features in the formation of the scrapie prion proteins. Proc. Natl. Acad. Sci..

[3] Safar J., Roller P.P., Gajdusek D.C., Gibbs C.J. (1993). Thermal stability and conformational transitions of scrapie amyloid (prion) protein correlate with infectivity. Protein Sci..

[4] Prusiner S.B. (1982). Novel proteinaceous infectious particles cause scrapie. Science.

[5] Willbold D., Strodel B., Schröder G.F., Hoyer W., Heise H. (2021). Amyloid-type protein aggregation and prion-like properties of amyloids. Chem. Rev..

[6] Kraus A., Hoyt F., Schwartz C.L., Hansen B., Artikis E., Hughson A.G. (2021). High-resolution structure and strain comparison of infectious mammalian prions. Mol. Cell.

[7] Wang L.-Q., Zhao K., Yuan H.-Y., Wang Q., Guan Z., Tao J. (2020). Cryo-em structure of an amyloid fibril formed by full-length human prion protein. Nat. Struct. Mol. Biol..

[8] Collinge J., Palmer M.S., Dryden A. (1991). Genetic predisposition to iatrogenic creutzfeldt-jakob disease. Lancet.

[9] Palmer M.S., Dryden A.J., Hughes J.T., Collinge J. (1991). Homozygous prion protein genotype predisposes to sporadic creutzfeldt–jakob disease. Nature.

[10] Baker H.E., Poulter M., Crow T.J., Frith C.D., Lofthouse R., Ridley R.M. (1991). Aminoacid polymorphism in human prion protein and age at death in inherited prion disease. Lancet.

[11] Baiardi S., Rossi M., Mammana A., Appleby B.S., Barria M.A., Cali I. (2021). Phenotypic diversity of genetic creutzfeldt–jakob disease: A histo-molecular-based classification. Acta Neuropathologica.

[12] Brown K., Mastrianni J.A. (2010). The prion diseases. J. Geriatr. Psychiatry Neurol..

[13] Barducci A., Chelli R., Procacci P., Schettino V., Gervasio F.L., Parrinello M. (2006). Metadynamics simulation of prion protein: _β_-Structure stability and the early stages of misfolding. J. Am. Chem. Soc..

[14] Hosszu L.L., Wells M.A., Jackson G.S., Jones S., Batchelor M., Clarke A.R. (2005). Definable equilibrium states in the folding of human prion protein. Biochemistry.

[15] Gerum C., Silvers R., Wirmer-Bartoschek J., Schwalbe H. (2009). Unfolded-state structure and dynamics influence the fibril formation of human prion protein. Angew. Chem..

[16] Ziaunys M., Sneideris T., Smirnovas V. (2020). Formation of distinct prion protein amyloid fibrils under identical experimental conditions. Scientific Rep..

[17] Torrent J., Martin D., Noinville S., Yin Y., Doumic M., Moudjou M. (2019). Pressure reveals unique conformational features in prion protein fibril diversity. Scientific Rep..

[18] Hosszu L.L., Trevitt C.R., Jones S., Batchelor M., Scott D.J., Jackson G.S. (2009). Conformational properties of *β*-prp. J. Biol. Chem..

[19] Greenfield N.J. (2006). Using circular dichroism spectra to estimate protein secondary structure. Nat. Protoc..

[20] Pfleger C., Rathi P.C., Klein D.L., Radestock S., Gohlke H. (2013). Constraint network analysis (cna): A python software package for efficiently linking biomacromolecular structure, flexibility,(thermo-) stability, and function. J. Chem. Inf. Model..

[21] Rathi P.C., Jaeger K.-E., Gohlke H. (2015). Structural rigidity and protein thermostability in variants of lipase a from bacillus subtilis. PLoS One.

[22] Hermans S.M., Pfleger C., Nutschel C., Hanke C.A., Gohlke H. (2017). Rigidity theory for biomolecules: Concepts, software, and applications. Wiley Interdiscip. Rev. Comput. Mol. Sci..

[23] Valley C.C., Cembran A., Perlmutter J.D., Lewis A.K., Labello N.P., Gao J. (2012). The methionine-aromatic motif plays a unique role in stabilizing protein structure. J. Biol. Chem..

[24] Lee J., Chang I. (2019). Structural insight into conformational change in prion protein by breakage of electrostatic network around h187 due to its protonation. Scientific Rep..

[25] Zhou S., Shi D., Liu X., Yao X., Da L.-T., Liu H. (2019). ph-induced misfolding mechanism of prion protein: insights from microsecondaccelerated molecular dynamics simulations. ACS Chem. Neurosci..

[26] Malevanets A., Chong P.A., Hansen D.F., Rizk P., Sun Y., Lin H. (2017). Interplay of buried histidine protonation and protein stability in prion misfolding. Scientific Rep..

[27] Baldwin A.J., Knowles T.P.J., Tartaglia G.G., Fitzpatrick A.W., Devlin G.L., Shammas S.L. (2011). Metastability of native proteins and the phenomenon of amyloid formation. J. Am. Chem. Soc..

[28] Hosszu L.L., Jackson G.S., Trevitt C.R., Jones S., Batchelor M., Bhelt D. (2004). The residue 129 polymorphism in human prion protein does not confer susceptibility to creutzfeldt-jakob disease by altering the structure or global stability of PrPC ∗. J. Biol. Chem..

[29] Hosszu L.L.P., Conners R., Sanger D., Batchelor M., Sawyer E.B., Fisher S. (2020). Structural effects of the highly protective v127 polymorphism on human prion protein. Commun. Biol..

[30] Tahiri-Alaoui A., Gill A.C., Disterer P., James W. (1992). Methionine 129 variant of human prion protein oligomerizes more rapidly than the. Lancet.

[31] Eghiaian F., Daubenfeld T., Quenet Y., van Audenhaege M., Bouin A.-P., van der Rest G. (2007). Diversity in prion protein oligomerization pathways results from domain expansion as revealed by hydrogen/deuterium exchange and disulfide linkage. Proc. Natl. Acad. Sci..

[32] Engelke A.D., Gonsberg A., Thapa S., Jung S., Ulbrich S., Seidel R. (2018). Dimerization of the cellular prion protein inhibits propagation of scrapie prions. J. Biol. Chem..

[33] Zahn R., Liu A., Lührs T., Riek R., von Schroetter C., Garcia F.L. (2000). NMR solution structure of the human prion protein. Proc. Natl. Acad. Sci..

[34] Rösener N.S., Gremer L., Reinartz E., König A., Brener O., Heise H. (2018). A d-enantiomeric peptide interferes with heteroassociation of amyloid-*β* oligomers and prion protein. J. Biol. Chem..

[35] Schuck P., Perugini M.A., Gonzales N.R., Howlett G.J., Schubert D. (2002). Size-distribution analysis of proteins by analytical ultracentrifugation: Strategies and application to model systems. Biophysical J..

[36] Abskharon R.N.N., Giachin G., Wohlkonig A., Soror S.H., Pardon E., Legname G. (2014). Probing the n-terminal _β_-sheet conversion in the crystal structure of the human prion protein bound to a nanobody. J. Am. Chem. Soc..

[37] (2022).

[38] Honda R.P., Yamaguchi K.-i., Kuwata K. (2014). Acid-induced molten globule state of a prion protein. J. Biol. Chem..

[39] Izadi S., Anandakrishnan R., Onufriev A.V. (2014). Building water models: a different approach. J. Phys. Chem. Lett..

[40] Case D.A., Cheatham T.E., Darden T., Gohlke H., Luo R., Merz K.M. (2005). The amber biomolecular simulation programs. J. Comput. Chem..

[41] Tian C., Kasavajhala K., Belfon K.A.A., Raguette L., Huang H., Migues A.N. (2020). ff19sb: Amino-acid-specific protein backbone parameters trained against quantum mechanics energy surfaces in solution. J. Chem. Theor. Comput..

[42] Roe D.R., Cheatham T.E. (2013). Ptraj and cpptraj: Software for processing and analysis of molecular dynamics trajectory data. J. Chem. Theor. Comput..

[43] Jacobs D.J., Rader A.J., Kuhn L.A., Thorpe M.F. (2001). Protein flexibility predictions using graph theory. Proteins: Struct. Funct. Bioinformatics.

[44] Rathi P.C., Jaeger K.-E., Gohlke H. (2015). Structural rigidity and protein thermostability in variants of lipase a from bacillus subtilis. PloS one.

[45] Barlow D.J., Thornton J. (1983). Ion-pairs in proteins. J. Mol. Biol..

[46] Ryckaert J.-P., Ciccotti G., Berendsen H.J. (1977). Numerical integration of the cartesian equations of motion of a system with constraints: Molecular dynamics of n-alkanes. J. Comput. Phys..

[47] Hopkins C.W., Le Grand S., Walker R.C., Roitberg A.E. (2015). Long-time-step molecular dynamics through hydrogen mass repartitioning. J. Chem. Theor. Comput..

