# Supplementary material for: Met/Val129 polymorphism of the full-length human prion protein dictates distinct pathways of amyloid formation
Source: J Biol Chem. 2022 Aug 28;298(10):102430. doi: 10.1016/j.jbc.2022.102430 (PMC9513279; doi:10.1016/j.jbc.2022.102430)
Supplement: Supporting Information [file mmc1.docx]

# Supporting information

Met/Val129 polymorphism of the full-length human prion protein dictates distinct pathways of amyloid formation

Thomas Pauly^+,1,2^, Najoua Bolakhrif^+,1,2^, Jesko Kaiser^3^, Luitgard Nagel-Steger^1,2^, Lothar Gremer^1,2^, Holger Gohlke^3,4,5,6^, and Dieter Willbold^*,1,2^

1. Institute of Biological Information Processing (IBI-7: Structural Biochemistry) and JuStruct: Jülich Center of Structural Biology, Forschungszentrum Jülich, 52425 Jülich, Germany
2. Institut für Physikalische Biologie, Heinrich-Heine-Universität Düsseldorf, 40225 Düsseldorf, Germany
3. Institute for Pharmaceutical and Medicinal Chemistry, Heinrich-Heine-Universität Düsseldorf, 40225 Düsseldorf, Germany
4. Jülich Supercomputing Centre (JSC), Forschungszentrum Jülich GmbH, 52425 Jülich, Germany
5. Institute of Bio- and Geosciences (IBG-4: Bioinformatics), Forschungszentrum Jülich GmbH, 52425 Jülich, Germany

6 John von Neumann Institute for Computing (NIC), Forschungszentrum Jülich GmbH, 52425 Jülich, Germany

+ Authors contributed equally

* Corresponding author: d.willbold@fz-juelich.de

## Supporting methods

- 1. Molecular dynamics simulations
  2. Uv- absorbance measurement

1. Supporting results
   1. Different protein concentrations and GdnHCl concentrations reveal distinct amyloid kinetics
2. Supporting figures
   1. Figure S1 Final purity of huPrP constructs
   2. Figure S2 Destabilization of huPrP
   3. Figure S3 Concentration series of huPrP
   4. Figure S4 Comparison of different huPrP and GdnHCl concentrations
   5. Figure S5 Soluble fraction during amyloid formation
   6. Figure S6 Impact of unstructured N-terminal region on oligomerization
   7. Figure S7 Secondary structure content before and during amyloid formation
   8. Figure S8 A) Frequency distribution of dihedral angles in all replicas and B) appearance of the dihedral angle over time during one replica

## Supporting methods

## Molecular dynamics simulations

Molecular dynamics simulations were performed using the ”Particle Mesh Ewald” method to consider long-range interactions; the SHAKE algorithm was applied to bonds involving hydrogen atoms [46]. The time step during thermalization and equilibration was set to 2 fs with a direct-space, non-bonded cutoff of 9.0 Å; the time step during the production runs was set to 4 fs as hydrogen mass repartitioning was used with a direct-space, non-bonded cutoff off 8.0 Å [47]. First, 102,500 steps of steepest descent and conjugate gradient minimization were performed; during 2,500, 50,000, and 50,000 steps positional harmonic restraints with force constants of 5 kcal×mol^-1^×Å^-2^, 1 kcal×mol^-1^×Å^-2^, and 0 kcal×mol^-1^×Å^-2^, respectively, were applied to the protein atoms. Then, 50 ps of NVT-MD (constant number of particles, volume, and temperature) were performed to heat the system to 100 K, followed by 250 ps of NPT-MD (constant number of particles, volume, and temperature) simulations to heat the system to 300 K and to adjust the density of the simulation box to a pressure of 1 atm. During these steps, a harmonic potential with a force constant of 1 kcal×mol^-1^×Å^-2^ was applied to protein atoms. Thereafter, 300 ps of NVT-MD simulations were conducted. During the first 250 ps of this step, the harmonic restraints were gradually reduced to zero. Then, ten independent replica of MD simulation production runs of 1 µs length were performed for each polymorph. The starting temperature of each run was varied by a fraction of a Kelvin.

To ensure that the dihedral angle of the mutated side chain does not impact the outcome of the MD simulations, we analyzed the dihedral angle between the planes defined by N, CA, CB and CA, CB, CG1 during the MD simulations using CPPTRAJ [42] (Figure S8). As all favored dihedral angles are found in this analysis (180°, -60°, and 60°) and there are frequent exchanges between these states, indicating that the side-chain of V129 is in equilibrium, the starting conformation of valine at position 129 should not influence the results. To be noted, the MD simulations do not include the glycosylation of the protein, as all experiments were performed with unglycosylated huPrP.

## Uv-absorbance measurement

Uv absorbance of soluble fraction was measured for both huPrP(23-230) variants during amyloid formation using the UV-1900i UV-VIS-Spectrophotometer (Shimadzu, Kyōto, Japan). 15 µM huPrP with 0.5 M GdnHCl were used. Samples were incubated in 1.5 mL Lobind Eppendorf tubes, at 37 °C and continuous shaking at 300 rpm. After 1, 5, 19, 55, 96 and 120 h, 100µl was taken and centrifuged (30 minutes, 15.000 ×g, 25°C). The supernatant was used to measure the remaining monomer and small oligomer content. The measurement was performed in duplicates in a quartz glass cuvette with 1 mm path length.

## Supporting results

**Different protein concentration and GdnHCl concentrations reveal distinct amyloid kinetics**

Different conditions were investigated for the amyloid formation kinetics. Figure S2 shows a concentration series of different GdnHCl concentrations with 15 µM of huPrP(23-230) 129M variant. These results indicate that a certain amount of GdnHCl is needed for sufficient destabilization and initialization of amyloid formation. The amount should not be too high to enable seed formation. Additionally, a concentration series with relatively low concentrations of both variants was established with a constant ratio of GdnHCl (Figure S3). Figure S4 confirms the importance of a certain huPrP/GdnHCl ratio for the 129M variant, as the lowest protein concentration tested with the highest applied GdnHCl concentration shows a significantly delayed fluorescence increase. For the highest protein concentration, the amount of GdnHCl alters the fluorescence signal of the initial plateau as well as the time of increase and duration of the interim and final plateau.

## Supporting figures

Figure S1: **Final purity of huPrP constructs** Coomassie stained 15% Tris/Glycine SDS-PAGE of 3 µM huPrP. 1, huPrP(23-230) 129V; 2, huPrP(23-230) 129M; 3, huPrP(121-230) 129M; M, marker protein

Figure S2: **Destabilization of huPrP.** Amyloid formation kinetics monitored by ThT fluorescence of 15 µM huPrP(23-230) 129M variant in the presence of different GdnHCl concentrations at pH 2. huPrP(23-230) 129M without GdnHCl shows no amyloid formation (grey).

Figure S3: **Concentration series of huPrP.** (A) Amyloid formation kinetics monitored by ThT fluorescence of different huPrP(23-230) concentrations for the 129M variant and (B) 129V variant at pH 2. The ratio between protein and GdnHCl is constant at 1 to 33,333. huPrP(23-230) without GdnHCl shows no amyloid formation (grey).

Figure S4: **Comparison of different huPrP and GdnHCl concentrations.** Amyloid formation kinetics monitored by ThT fluorescence of 7.5 and 30 µM huPrP(23-230) for both variants in the presence of 0.25 and 1 M GdnHCl at pH 2.

Figure S5: **Soluble fraction during amyloid formation.** Absorbance spectra of 15 µM huPrP (23-230) 129M and 129V variant at pH 2. Spectra were measured before amyloid formation without GdnHCl and after 1, 5, 19, 55, 96 and 120 h during amyloid formation in the presence of 0.5 M GdnHCl. Left and middle graphs show the absorbance plotted against the wavelengths (in nm) for the 129M and the 129V variant, respectively. Right graph represents the relative absorbance (in %) plotted against the time (in h).

Figure S6: **Impact of the unstructured N-terminal region on oligomerization.** (A) Comparison of results from SV analysis of 7.5 µM of the 129M variant huPrP(121-230) (light blue) and huPrP(23-230) (dark blue) and the 129V variant huPrP(23-230) (orange) at pH 2. Raw data of the 129M variant huPrP(121-230) with fitted Lamm-equation solutions from *c*(*s*) model are color-coded for the duration of sedimentation. (B) The result of data fitting is an *s*-value distribution. The distributions of huPrP(23-230) for both variants are shown for comparison.

Figure S7: **Secondary structure content before and during amyloid formation.** CD spectra of 10 µM huPrP(23-230) 129M and 129V variant at pH 2. Spectra were measured before amyloid formation without GdnHCl and after 5 and 72 h during amyloid formation in the presence of 0.5 M GdnHCl.

Figure S8: **A)** **Frequency distribution of dihedral angles in all replicas** and **B) appearance of the dihedral angle over time during one replica**; the dihedral angle was determined between the planes spanned by N, CA, CB and CA, CB, CG1 during MD simulations.
